# Protocol: developing a conceptual framework of patient mediated knowledge translation, systematic review using a realist approach

**DOI:** 10.1186/1748-5908-6-25

**Published:** 2011-03-22

**Authors:** Anna R Gagliardi, France Légaré, Melissa C Brouwers, Fiona Webster, David Wiljer, Elizabeth Badley, Sharon Straus

**Affiliations:** 1 Departments of Health Policy, Management and Evaluation, University of Toronto, Toronto, Canada; 2 Department of Family Medicine, Université de Laval Centre Hospitalier, Universitaire de Québec, Québec, Canada; 3 Department of Oncology; Department of Clinical Epidemiology and Biostatistics, McMaster University, Hamilton, Canada; 4 Holland Orthopaedic & Arthritic Centre, Sunnybrook Health Sciences Centre, Toronto, Canada; 5 Department of Radiation Oncology, University Health Network; Faculty of Medicine University of Toronto, Toronto, Canada; 6 Department of Health Care and Outcomes Research and Epidemiology, University Health Network, Toronto, Canada; 7 Departments of Medicine, University of Toronto, Toronto, Canada

## Abstract

**Background:**

Patient involvement in healthcare represents the means by which to achieve a healthcare system that is responsive to patient needs and values. Characterization and evaluation of strategies for involving patients in their healthcare may benefit from a knowledge translation (KT) approach. The purpose of this knowledge synthesis is to develop a conceptual framework for patient-mediated KT interventions.

**Methods:**

A preliminary conceptual framework for patient-mediated KT interventions was compiled to describe intended purpose, recipients, delivery context, intervention, and outcomes. A realist review will be conducted in consultation with stakeholders from the arthritis and cancer fields to explore how these interventions work, for whom, and in what contexts. To identify patient-mediated KT interventions in these fields, we will search MEDLINE, the Cochrane Library, and EMBASE from 1995 to 2010; scan references of all eligible studies; and examine five years of tables of contents for journals likely to publish quantitative or qualitative studies that focus on developing, implementing, or evaluating patient-mediated KT interventions. Screening and data collection will be performed independently by two individuals.

**Conclusions:**

The conceptual framework of patient-mediated KT options and outcomes could be used by healthcare providers, managers, educationalists, patient advocates, and policy makers to guide program planning, service delivery, and quality improvement and by us and other researchers to evaluate existing interventions or develop new interventions. By raising awareness of options for involving patients in improving their own care, outcomes based on using a KT approach may lead to greater patient-centred care delivery and improved healthcare outcomes.

## Background

Knowledge translation (KT) refers to an iterative approach for improving healthcare delivery, utilization, and outcomes by synthesizing pertinent research, interacting with users to identify needs and barriers, employing tailored strategies to promote adoption of evidence-based recommendations, and evaluating or monitoring their impact [1]. A patient-centred health system is responsive to patient needs and values and places patients in the centre of this system [2]. It recognizes that communication with, active involvement of, and attributes or circumstances of patients mediate the trajectory from care delivery to optimal outcomes [3]. Engaging patients in their own healthcare may have considerable potential to achieve beneficial outcomes [4-6]. One systematic review found that education for diabetic patients, counseling for lifestyle modification among mental health patients, and reminders for cancer screening tests had a moderate to large effect on treatment compliance and outcomes [7]. However, it is difficult to draw conclusions because studies targeting patients were few, and strategies varied by intent, format, and type of patient or clinical context. Similarly, an international group of rheumatologists issued recommendations for the purpose (self-management, treatment compliance), format (oral plus print, group education, self-help groups), and content (knowledge and management, drug side effects) of patient education interventions, but found evidence to be sparse [8]. A comprehensive understanding of the range of interventions and their underlying mechanisms and impact is needed to guide future efforts that develop, implement, and/or evaluate the cost-effectiveness of patient-mediated KT interventions.

## Proposed Theoretical Framework

**Figure 1:**
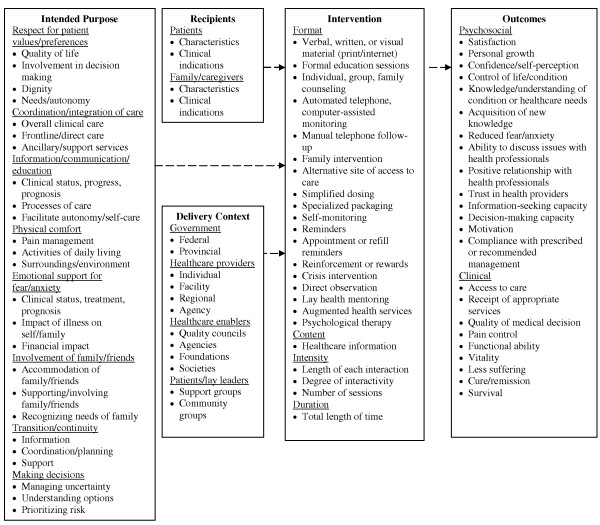
**Conceptual framework of patient-mediated knowledge-translation interventions**.

We compiled a preliminary conceptual framework of patient-mediated KT from several sources (Figure 1). A concept analysis defined contexts that provide patients with participatory opportunities, including interactions with healthcare providers in different settings and through programs offered by various types of agencies [9]. Evaluation of information needs among arthritis patients, and of the UK Health in Partnership program, results identified potential outcomes, including both psychosocial and clinical benefits (5,10). A population-based survey of patients and health professionals in the United States identified patient-centred care dimensions analogous to patient-mediated KT intent, including respect for patient values, coordination and continuity of care, education, physical comfort, emotional support, decision making, and involvement of family and friends [11]. A concept analysis on the association of patient communication with improved health offered both intent and outcomes results [3]. A Cochrane review on strategies to promote medication compliance described a wide range of intervention formats [12]. This framework serves as a starting point, and hypothetical linkages between components will be confirmed, expanded, and refined through the proposed study.

## Purpose

The purpose of this study is to develop a conceptual framework of patient-mediated KT interventions by synthesizing knowledge on type of intervention (format, content), outcome (intended, reported), mechanism of action (reported or applicable theory), and application (clinical indication, healthcare setting, attributes of those delivering and receiving care) based on a review of the relevant literature.

## Methods

### Approach

Knowledge synthesis is often required to describe what is known on a particular topic and identify the need for further research. We will conduct a realist review based on the methods described by Pawson *et al*. [13]. This five-step approach was developed to explore how complex interventions work for whom and in what contexts. A realist review focuses on describing theoretical and contextual details about why the intervention did or did not work that can be used to fine-tune its design. It draws upon a wide range of quantitative and qualitative study designs. This approach was recently used to examine the interaction between context, intervention, outcome, and underlying theory to understand the efficacy of school feeding programs [14].

To be feasible, realist reviews must be bounded by focusing the question on either particular processes or groups of recipients; thus, we will restrict our review to arthritis and cancer. Both represent prevalent conditions that have generated considerable research on patient involvement through education and self-management, but systematic reviews revealed variable impacts of these outcomes and called for further investigation of the factors that influence their effectiveness [15-17]. By focusing on two conditions, the review will be limited to a manageable number of studies, while still allowing for comparison by patient and contextual factors.

### Step 1: clarify scope--refine purpose of review/key theories to be explored

To refine the research questions and theories of interest, we will consult with stakeholders, including arthritis and cancer researchers, clinicians, managers, and patients, after first conducting an exploratory scan of the literature. A useful starting point is provided by another realist-inspired analysis in which 26 behaviour-change techniques were identified in a Cochrane Library review of interventions to promote physical activity [6]. In this study the taxonomy was validated by using it to code strategies and associated theories in a review of studies to encourage healthy eating. Additional theories relevant to patient-mediated KT will be assembled by searching indexed sources of literature, including MEDLINE and CINAHL, for [(models, theoretical or models, educational or models, psychological) AND patient education as topic or (information dissemination and patient participation)]. The research team will review the assembled theories to refine review questions, guide the selection of relevant theories, and confirm or expand the conceptual framework upon which a more comprehensive literature review will be based.

### Step 2: search for evidence

A comprehensive literature search developed by an information specialist will be conducted by using several indexed sources. Search strategies will combine concepts reflecting [(arthritis or neoplasms) AND patient education as topic or (information dissemination and patient participation)]. Searches will be executed for the years 1995 to current to encompass a nearly 15-year span during which research on patient involvement became prevalent. Databases include MEDLINE (North American), the Cochrane Library (systematic reviews, trials), EMBASE (European), and CINAHL (nursing, allied health). To augment these searches, we will examine five years of tables of contents for journals likely to publish patient-mediated KT interventions, including *Patient Education and Counseling*, *Health Expectations*, *Implementation Science*, *Journal of Cancer Education*, *Psycho-Oncology*, *Arthritis Care & Research*, and *Communication & Medicine*. To ensure that all relevant literature is captured, we will scan the references of eligible studies. Quantitative (meta-analyses, systematic reviews, guidelines, surveys, observational studies, randomized trials) or qualitative (interviews, focus groups) studies published in the English language that focus on developing, implementing, or evaluating patient-mediated KT interventions are eligible. Abstracts, letters, or editorials are ineligible. Two individuals will independently review titles and abstracts and select articles for inclusion based on eligibility criteria using a screening tool. Articles selected by at least one reviewer will be retrieved since ultimate judgment about inclusion must be reserved until the full text is examined.

### Step 3: extract data and appraise primary studies

A data-extraction form will be developed based on the refined version of the conceptual framework (Step 1) to collect information on intervention (format, content), outcome (intended, reported), mechanism of action (theory explicitly reported by authors or referred to implicitly in objectives or methods), and application (clinical indication, setting of care, attributes of those delivering and receiving the intervention). As a pilot, data will be extracted independently by the principal investigator and research associate for 10 randomly selected articles. They will compare congruence of extracted data and determine whether and how the form should be revised, then independently extract data from remaining studies. Most details will be noted on the form by checking the appropriate box. Qualitative details, including description of implicit theory, will be highlighted in the article, which will be copied and attached to the data extraction form. Study quality will be assessed using criteria relevant to study design to describe the nature of this literature but will not be used to exclude studies from review [18-20].

### Step 4: synthesize and interpret

The research associate will tabulate extracted/highlighted quantitative and qualitative data, noting any differences between independently extracted information for the same article and resolving those differences through discussion with the principal investigator. The total number of eligible and included studies from each source will be reported, along with reasons for exclusions. Tabulated findings will be examined to discuss the quantity, design, and quality of studies. The nature of patient-mediated KT interventions will be described according to the elements of the refined conceptual framework, including purpose, context, recipient characteristics, intervention design and delivery, explicit theory, and outcomes. Contextual information will be further examined thematically according to May's narrative review approach [21]. This involves directly summarizing relevant details as they are reported to identify recurring or important issues, without any attempt to transform them into a common metric or interpreted theme as in a standard qualitative analysis. Qualitative details will be independently examined by the principal applicant and research associate. They will compare findings and resolve differences through discussion. Findings will be summarized to describe interventions, how they work, for whom, and in what context and identify explicit and implicit theories relevant to interventions. These data will be used to expand the conceptual framework and to create a separate taxonomy of patient-mediated KT strategies and associated relevant theories.

The research team will review and interpret the findings and confirm or further refine the products, which include the following: (a) a conceptual framework of patient-mediated KT interventions and outcomes; (b) a description of patient-mediated KT interventions and the degree to which they have been evaluated in different settings or patients, highlighting key elements of design or implementation that contribute to or detract from their impact; (c) a taxonomy listing the variety of patient-mediated KT interventions and associated theories; (d) recommendations for systematic review of particular patient-mediated KT interventions where evidence is found to be sufficient; and/or (e) identification of research gaps that warrant further investigation through primary research study by comparing findings and the nature/quality of that evidence to concepts in the conceptual framework.

## Discussion

Evidence suggests that informing, educating, and supporting patients to engage in their own healthcare leads to improved utilization and outcomes. Preliminary examination of syntheses of this research highlights that we lack information on how best to deliver patient-mediated KT interventions. We will examine this issue by comprehensively reviewing and synthesizing the available literature in partnership with decision makers/users with the responsibility for engaging patients and caregivers. Several products or outcomes are anticipated. A knowledge synthesis of patient-mediated KT interventions will result in a deeper understanding of how differing design, delivery, and context influence their impact. Along with the conceptual framework of patient-mediated KT options and outcomes, this information could be used by healthcare providers, managers, educationalists, patient advocates, and policy makers to guide program planning, service delivery, and quality improvement. We and other researchers can use both the conceptual framework and taxonomy associating relevant theories with patient-mediated KT strategies to evaluate existing interventions or develop new interventions. Ensuing research may be more useful because interventions could be operationalised using a common approach, and the factors contributing to success or failure could be more thoroughly elucidated and considered in intervention design. Gaps in knowledge will be identified, which may lead to the development of novel forms of patient-mediated KT interventions or the testing of existing strategies in unique contexts. Ultimately, by raising awareness of the range and nature of options for involving patients in improving their own healthcare outcomes based on contextualising this literature using a KT approach, influencing practical development of patient-mediated KT strategies by individual and organizational providers, and driving further research in this area, this knowledge may lead to greater patient-centred care delivery and improved healthcare outcomes.

## Competing interests

The authors declare that they have no competing interests.

## Authors' contributions

ARG and FL conceptualised and designed this study, prepared the proposal, and obtained funding. ARG will lead and coordinate data collection and analysis, interpretation, and report writing. She will be the primary investigator to independently review and extract data from articles and manuscripts. All investigators contributed to design of the study through several meetings, teleconferences, and email correspondences. FL and SS will oversee the study as research mentors to ARG. FL, FW, and EB will all contribute to planning, interpretation, report writing, and dissemination from the arthritis perspective. MCB, DW, and SS will all contribute to planning, interpretation, report writing, and dissemination from the cancer perspective. All investigators will assist in identifying and engaging relevant decision makers as well as assisting with interpretation, report writing, and dissemination activities. All investigators read and approved the final version of his manuscript.
